# Relative Risk of Peripheral Neuropathy With Ado-Trastuzumab Emtansine (T-DM1) Compared to Taxane-Based Regimens in Human Epidermal Growth Factor Receptor 2 (HER2)-Positive Cancers: A Systematic Review and Meta-Analysis

**DOI:** 10.7759/cureus.15282

**Published:** 2021-05-27

**Authors:** Nusrat Jahan, Shabnam Rehman, Rafiullah Khan, Catherine Jones

**Affiliations:** 1 Division of Hematology and Oncology, Department of Internal Medicine, Texas Tech University Health Sciences Center, Lubbock, USA; 2 Division of Hematology and Oncology, Department of Internal Medicine, University of Cincinnati Medical Center, Cincinnati, USA

**Keywords:** ado-trastuzumab emtansine, t-dm1, docetaxel, paclitaxel, taxane, peripheral neuropathy, peripheral sensory neuropathy, trastuzumab emtansine

## Abstract

Background

Peripheral neuropathy (PN), especially peripheral sensory neuropathy (PSN), is significant toxicity of taxanes, the most used class of microtubule inhibitors for human epidermal growth factor receptor 2 (HER2)-positive breast cancer patients. Ado-trastuzumab emtansine (T-DM1) is a HER2-targeted antibody-drug conjugate, consisting of trastuzumab and a microtubule inhibitor DM1, which has been approved for HER2-positive breast cancer. T-DM1 has also been found to cause significant PN, including PSN.

Methods

We conducted a systematic review and meta-analysis of phase 3 randomized controlled trials using T-DM1 in the experimental arm and a taxane-based regimen in the control arm to determine the relative risk of PN and PSN associated with T-DM1 as compared to taxanes. A total of 1,857 patients were included in the analysis. The Cochran-Mantel-Haenszel method and the random-effects model were used to calculate the pooled risk ratio (RR) with a 95% confidence interval (CI) for all-grade and grade ≥3 PN and PSN.

Results

The relative risks of all-grade PN and all-grade PSN were lower with T-DM1 compared to taxanes. The pooled RR of all-grade PN was 0.59, 95% CI: 0.39-0.89, P = 0.01, and the pooled RR of all-grade PSN was 0.58, 95% CI: 0.46-0.74, P < 0.0001.

Conclusions

Our meta-analysis demonstrated that T-DM1 is associated with a relatively lower risk of all-grade PN and PSN than the taxane-based regimens for HER2-positive cancers. It could be an area of consideration in selecting therapy for HER2-positive breast cancer patients at high risk of developing or having pre-existing PN and PSN.

## Introduction

Human epidermal growth factor receptor 2 (HER2)-positive breast cancers (BCs) account for approximately 15%-20% of the BCs diagnosed in the United States [1-2]. Before the advent of HER2-targeted therapies, HER2 overexpression was regarded as a poor prognostic marker for BC and was associated with worse outcomes [3]. The discovery of HER2-targeted therapy was a paradigm shift in the management of HER2-positive BC. Ado-trastuzumab emtansine (T-DM1) was the first HER2-targeted antibody-drug conjugate to get the approval of the Food and Drug Administration (FDA) in 2013 for the treatment of metastatic HER2-positive BC. T-DM1 consists of a humanized anti-HER2 monoclonal antibody trastuzumab and a small maytansinoid microtubule inhibitor, DM1. T-DM1 binds to sub-domain IV of the HER2 receptor through trastuzumab and ensures selective delivery of DM-1 into the HER2-overexpressing cells. The DM-1, a potent microtubule inhibitor, causes cell cycle arrest, mitotic catastrophe, disruption of intracellular trafficking, and apoptosis [4-5]. Despite the conjugation with DM-1, the trastuzumab portion of T-DM1 continues to exhibit HER2-receptor-mediated pathway inhibition and antibody-dependent cell-mediated cytotoxicity [4]. Currently, T-DM1 is approved for the metastatic HER2-positive BC patients at the second line after receiving trastuzumab and taxanes, and for the HER2-positive early BC patients at the adjuvant setting who have residual invasive disease after neoadjuvant treatment with a taxane and trastuzumab-based regimen [6]. T-DM1 has some unique adverse effects, including thrombocytopenia, hemorrhage, peripheral neuropathy, hepatotoxicity, and pulmonary toxicity [6]. Dose-dependent axonal degeneration was observed in preclinical studies of T-DM1, which was not reversible after six weeks of observation [4]. In different phase 3 clinical trials, the incidence of T-DM1 induced peripheral neuropathy (PN) was between 3% and 28%, and peripheral sensory neuropathy (PSN) was between 4% and 18.6% [6-8].

Taxanes such as paclitaxel and docetaxel are the most commonly used microtubule inhibitors for BC patients. The overall incidence of PN associated with paclitaxel is between 57% and 83%, and docetaxel is between 11% and 64% [9]. Several small studies have reported that obesity, increased body surface area, advanced age, diabetes, poor nutritional status, and low hemoglobin level are some risk factors for taxane-induced PN [10-13]. We conducted a systematic review and meta-analysis of phase 3 randomized controlled trials (RCTs) using T-DM1 in the experimental arm and a taxane-based regimen in the control arm to determine the relative risk of PN and PSN associated with T-DM1 compared to taxanes.

## Materials and methods

We performed our literature search in the PubMed, EMBASE, American Society of Clinical Oncology (ASCO) meeting abstracts, and San Antonio Breast Cancer Symposium meeting abstracts from inception through April 30, 2020, using the following keywords: 'ado-trastuzumab emtansine,' 'trastuzumab emtansine,' and 'T-DM1.' We limited our search to 'human' and 'English.' After removing duplicates, all the abstracts were screened to identify eligible studies. Furthermore, we reviewed references of relevant studies for any additional studies. Eligibility criteria used for study selection were: (1) Phase 3 RCTs used T-DM1 in the intervention arm and a taxane in the control arm, and (2) Reported the number of events for all-grade PN and PSN for both the intervention and control arms. For eligibility assessment and data extraction, at least two authors screened each study. Disagreements were resolved by building consensus among all authors. For analysis, we included the safety population defined as the patients who received at least one dose of the study drug. We followed Preferred Reporting Items for Systematic Reviews and Meta-Analyses (PRISMA) guidelines for search, study selection, and analysis in this study [14].

The Cochran-Mantel-Haenszel method and the random-effects model were used to calculate the pooled risk ratio (RR) for all-grade and grade ≥3 PN and PSN with a 95% confidence interval (CI). An RR of < 1 was considered favorable for T-DM1, and an RR of >1 was considered unfavorable for T-DM1. A P-value of ≤0.05 was considered statistically significant. Heterogeneities across the studies were assessed by Cochran’s Q test and I^2^ value. An I^2^ value of > 50% was considered substantially heterogeneous. All statistical analyses were performed with the Review Manager, version 5.3 (Nordic Cochrane Centre, Copenhagen, Denmark). Biases of the studies were assessed using the Cochrane Collaboration’s tool for bias assessment [15].

## Results

**Figure 1:**
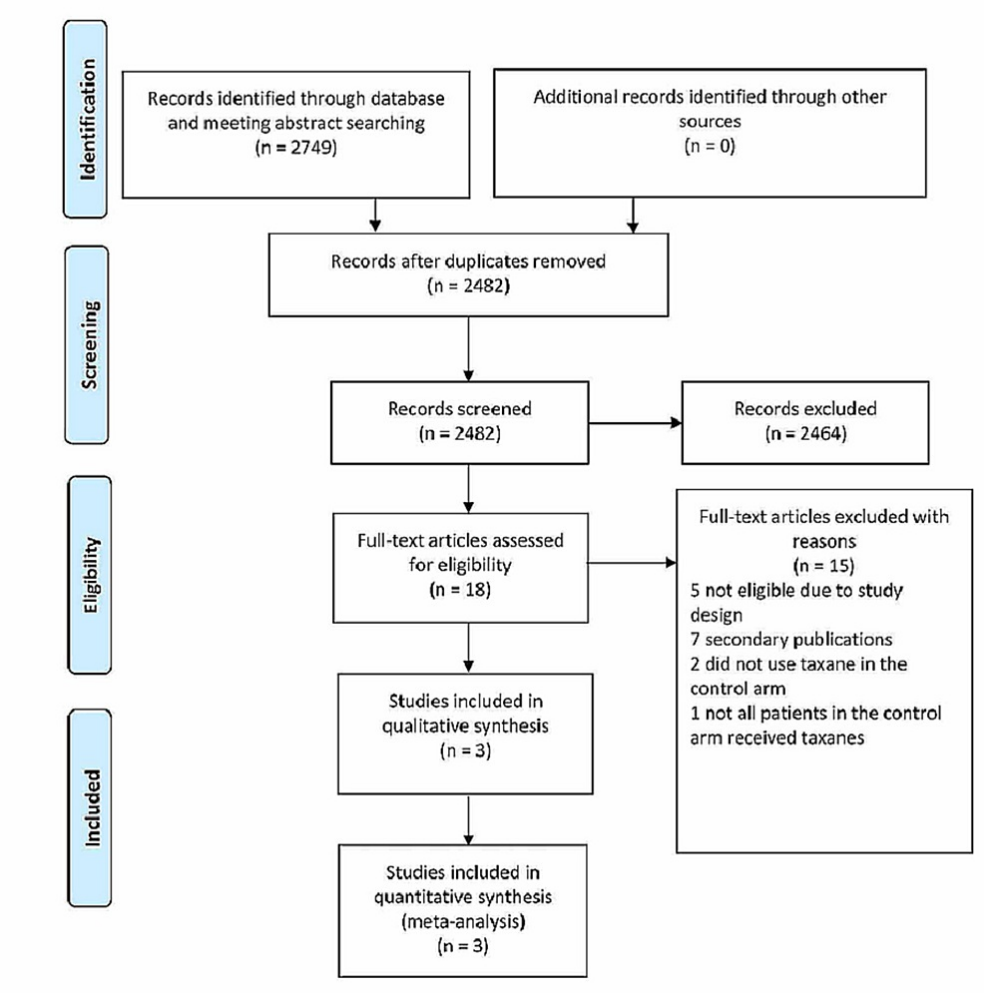
PRISMA flow diagram of the study selection process PRISMA: Preferred Reporting Items for Systematic Reviews and Meta-Analyses

According to our search strategies described in the method section, we initially identified 2,749 references. After removing 267 duplicates, we reviewed 2,482 abstracts to identify potential studies. At the initial screening, we excluded 2,464 abstracts. The most common reasons for excluding abstracts were not a phase 3 RCT, conference papers, or secondary publications. Eighteen full-text articles were reviewed, and 10 potential trials were identified for final consideration. After a meticulous assessment, we concluded that only three phase 3 RCTs, the GATSBY, KRISTINE (neoadjuvant phase), and MARIANNE trials, met both the inclusion criteria of our meta-analysis and were included in the final analysis [7,16-17]. Figure 1 depicts the details of the study selection process. These trials randomized 1,857 participants: 1,174 in the T-DM1 arms and 683 in the taxane arms.

The KRISTINE and MARIANNE trials were conducted in BC patients, and the GATSBY trial was done in gastric and gastroesophageal junction adenocarcinoma patients [7,16-17]. In the experimental arm, T-DM1 +/- pertuzumab was used in all three studies. In the control arm, KRISTINE used carboplatin, docetaxel, trastuzumab, and pertuzumab; MARIANNE used taxanes and trastuzumab; the GATSBY used taxanes. The dose of T-DM1 was 3.6 mg/kg every three weeks in the MARIANNE and KRISTINE trials and 2.4 mg/kg every week in the GATSBY trial. In three studies, the dose of docetaxel varied between 75 and 100 mg/m^2^ every three weeks, and the dose of paclitaxel was 80 mg/m^2^ every week. The median duration of T-DM1 use was 7.7 weeks, 18 weeks, and 45 weeks in the GATSBY, KRISTINE (neoadjuvant phase), and MARIANNE trials, respectively. Whereas, in the control arm, the median duration taxane use was 8.5 weeks for docetaxel and 12 weeks for paclitaxel in the GATSBY, 18 weeks in the KRISTINE (neoadjuvant phase), and 21 weeks in the MARIANNE trials. Some essential characteristics of these studies, such as the regimen used, the dose of medications, and the median duration of treatment, are included in Table 1. The severity of PN and PSN were reported according to the National Cancer Institute Common Terminology Criteria for Adverse Events (NCI CTCAE), version 4.0, in the KRISTINE and MARIANNE trials, and according to the NCI CTCAE, version 4.03, in the GATSBY trial.

**Table 1:** Characteristics of the studies included in the meta-analysis GE: gastroesophageal; RCT: randomized controlled trial; T-DM1: ado-trastuzumab emtansine; wk: week; (*): planned dose

Study	First author, year of publication	Study phase, type	Number of arms	Cancer type and line of treatment	Number of patients		Regimen used		T-DM1 dose*	Taxane dose*	The median duration of treatment	
					T-DM1 arm	Control arm	T-DM1 arm	Control arm			T-DM1	Taxane
GATSBY [16]	Thuss-Patience, 2017	2/3, RCT	2	Advanced gastric or GE junction adenocarcinoma after first-line therapy	224	111	T-DM1	Docetaxel or paclitaxel	2.4 mg/kg every wk	Docetaxel 75 mg/m^2 ^every 3 wks, or paclitaxel 80 mg/m^2 ^every wk	7.7 wks	Docetaxel: 8.5 wks, paclitaxel: 12 wks
KRISTINE [7]	Hurvitz, 2018	3, RCT	2	Stage II-III breast cancer at neoadjuvant setting	223	219	T-DM1 + pertuzumab	Docetaxel + carboplatin + trastuzumab + pertuzumab	3.6 mg/kg every 3 wks	Docetaxel 75 mg/m^2 ^every 3 wks	18 wks	18 wks
MARIANNE [17]	Perez, 2016	3, RCT	3	Advanced breast cancer at first-line setting	727 (T-DM1: 361, T-DM1 + pertuzumab: 366)	353	T-DM1 +/- pertuzumab	Docetaxel or paclitaxel + trastuzumab	3.6 mg/kg every 3 wks	Docetaxel 75 - 100 mg/m^2 ^every 3 wks, or paclitaxel 80 mg/m^2 ^every wk	45 wks	21 wks

**Figure 2:**
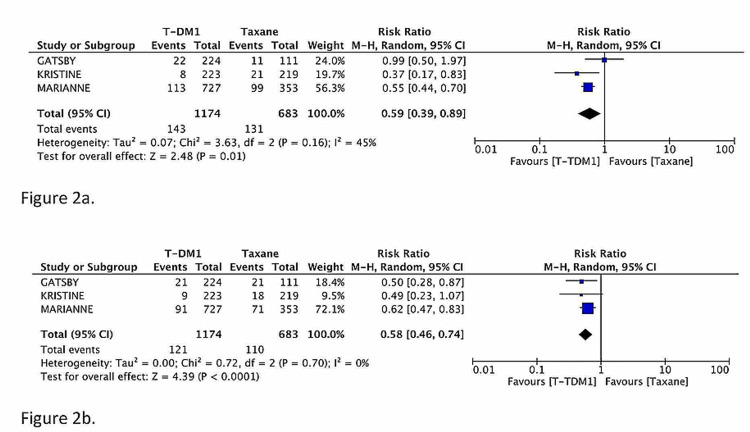
Pooled risk ratio (RR) of all-grade peripheral neuropathy (Figure 2a) and all-grade peripheral sensory neuropathy (Figure 2b) associated with T-DM1 compared to taxane-based regimens. The Cochran-Mantel-Haenszel method and the random-effects model were used to calculate the pooled RR. Statistical analyses were performed with Review Manager, version 5.3 (Nordic Cochrane Centre, Copenhagen, Denmark).

The incidences of all-grade PN in the T-DM1 arm and taxane arm of included studies were 12.1% and 19.2%, respectively. The relative risk of all-grade PN was significantly lower with T-DM1 as compared to taxane (pooled RR: 0.59, 95% CI: 0.39-0.89, P = 0.01, I^2^ = 45%) (Figure 2a). The incidences of all-grade PSN in the T-DM1 arm and taxane arm were 10.3% and 16.1%, respectively. The relative risk of all-grade PSN was also significantly lower with T-DM1 compared to taxane (pooled RR: 0.58, 95% CI: 0.46-0.74, P < 0.0001, I^2^ = 0%) (Figure 2b).

The MARIANNE trial has not reported the number of events for grade ≥3 PN and PSN. In GATSBY and KRISTINE, the incidence of grade ≥3 PN for T-DM1 was 1.6%, and it was 0.3% for taxane; this difference was not statistically significant (pooled RR: 1.70, 95% CI: 0.08-38.32, P = 0.74, I^2^ = 53%). The incidence of grade ≥3 PSN in GATSBY and KRISTINE for T-DM1 was 1.1% and for taxane, it was 1.8%; the difference was also not statistically significant (pooled RR: 0.59, 95% CI: 0.09-4.04, P = 0.59, I^2^ = 37%).

## Discussion

Taxane-induced PN and PSN are significant dose-limiting toxicities for patients receiving taxane-based chemotherapy for BC [18-19]. Our meta-analysis has demonstrated that all-grade PN and PSN incidence and risk are significantly lower with T-DM1 containing regimens than taxane-based regimens for HER2-positive cancers. However, there are several limitations in our study: (1) We were able to include only three studies meeting our inclusion criteria. (2) The risk of grade 3 and higher PN and PSN was not significantly different between the groups. We suspect it is due to the limited number of included studies and the different T-DM1 dosing schedules used in the GATSBY trial (2.4 mg/kg weekly). (3). Our meta-analysis was performed with aggregated data. We did not have access to some critical individual participant data, including the rate of discontinuation of treatment due to PN and PSN, the timeline of development, and the progression of PN and PSN in these studies. (4) The included studies were conducted in the different patient populations such as early-stage and advanced breast cancer and advanced gastric/gastroesophageal cancer. However, we do not think this has impacted our findings significantly. Our study aimed to compare the risk of neurotoxicity associated with T-DM1 and taxane use, not to compare the efficacy of these medications. (5) We also did not have information about the incidence of preexisting neuropathies and the tolerance of T-DM1 and taxanes in that group of patients. (6) All three studies used the NCI CTCAE for reporting PN and PSN that provided homogeneity in our meta-analysis. However, the assessment of PN and PSN by the NCI CTCAE is a clinician-based grading system, not an objective measure of nerve damage, and tends to underestimate chemotherapy-induced PN and PSN, especially in the earlier stages of chemotherapy [20-21].

We included data of the KRISTINE trial from the initial publication of the neoadjuvant phase instead of the most recent publication, including data from both the neoadjuvant and adjuvant phases [7,22]. In KRISTINE, T-DM1 was compared with a taxane-based regimen only in the neoadjuvant phase. T-DM1 and pertuzumab were continued in the study arm in the adjuvant phase, whereas the control arm received trastuzumab and pertuzumab. Hence, we concluded it was more appropriate to include data from the neoadjuvant phase rather than from both phases. Here, it is worth mentioning that T-DM1-induced PN and PSN seem to be cumulative. The incidences of PN and PSN increase with the increasing number of administering doses [8,22-23].

Currently, T-DM1 is FDA approved for two indications, as a second-line treatment of HER2-positive metastatic BC and as an adjuvant treatment of HER2-positive early BC with residual invasive cancer after neoadjuvant treatment [6]. The findings of our study may help individualize treatment for other HER2-positive BC patients based on the individual risk of PN and PSN. For example, the final results of the MARIANNE trial have established that T-DM1 is equally effective as the taxane and trastuzumab combination in the first-line setting for metastatic HER2-positive BC [24]. Therefore, in patients with a high risk of developing, or with pre-existing, PN and PSN, T-DM1 could be used as a first-line treatment. In the MARIANNE trial, T‐DM1 using arms reported longer median times to a clinically meaningful deterioration of neuropathic symptoms [24]. Although multiple studies have explored the potential roles of T-DM1 in the treatment of HER2 expressing gastric, gastroesophageal, colorectal, lung, and other cancers, it has not been approved for any non-breast malignancies yet [6,16,25-28]. Therefore, we have focused our discussion primarily on the use of T-DM1 in HER2-positive breast cancer.

## Conclusions

In conclusion, the lower risk of neuropathy associated with T-DM1 than taxanes, especially when T-DM1 is used for a limited duration, is an important and clinically relevant finding. T-DM1 is a promising medication in the treatment of breast cancer. Multiple trials are currently underway exploring several potential future usages of T-DM1 for HER2-positive breast and non-breast cancers. For example, for breast cancer, the KAITLIN trial (NCT01966471) and the ATEMPT trial (NCT01853748) in the adjuvant setting; and the TEAL trial (NCT02073487) in the neoadjuvant setting; the HER2CLIMB-02 trial (NCT03975647); and NCT03530696 in the advanced setting. Besides, several trials are exploring the efficacy of T-DM1 in various non-breast HER2-positive cancers, such as NCT04620187: postoperative or adjuvant treatment of HER2-positive salivary gland carcinomas, NCT03784599: trastuzumab-emtansine/osimertinib combination therapy in patients with advanced EGFR mutation-positive non-small-cell lung cancer with HER2 bypass track resistance, NCT02675829: HER2 amplified or mutant solid tumors, and more. A favorable neurotoxic profile of T-DM1 will be reassuring to the clinicians. However, future prospective studies are warranted to define the T-DM1-induced PN and PSN better.
